# Supplementary material for: Stimulation of Arabidopsis thaliana Seed Germination at Suboptimal Temperatures through Biopriming with Biofilm-Forming PGPR Pseudomonas putida KT2440
Source: Plants (Basel). 2024 Sep 24;13(19):2681. doi: 10.3390/plants13192681 (PMC11479300; doi:10.3390/plants13192681)
Supplement: Supplementary file 1 [file plants-13-02681-s001.zip › plants-3108016-supplementary.pdf]

Supplementary Table S1. Polynomial regression equations from Figure 2 used to calculate the optimal temperature, representing the peak of the polynomial curve.

	**Polynomial Equation**	**R^2**	**Optimal Temperature (°C)**
Col0	$y= - 0.62368\ x^2 + 24.45217\ x - 164.704$	0.99	19.6
Cvi	$y= -0.47284\ x^2 + 18.53673\ x - 114.497$	0.95	19.6
Bur	$y= -0.34765\ x^2 + 14.35104\ x - 73.8053$	0.77	20.6
C24	$y= -0.39764\ x^2 + 14.04123\ x -77.0408$	0.88	17.7
Bla1	$y= -0.36769\ x^2 + 12.94516\ x -44.3925$	0.94	17.6
Neo6	$y= -0.32623\ x^2 + 11.87753\ x -48.5092$	0.99	18.2
N13	$y= -0.83367\ x^2 + 33.691\ x -244.169$	0.98	20.2
Dja1	$y= -0.43448\ x^2 + 16.29822\ x -98.0474$	0.99	18.7
MS0	$y= -0.22952\ x^2 + 8.172436\ x -46.5713$	0.76	17.8
